# Long-Term Impact of Early-Life Stress on Hippocampal Apoptotic Gene Expression in *BALB/c* Mice

**DOI:** 10.1007/s11064-026-04822-7

**Published:** 2026-06-25

**Authors:** Aida Nurul Barokah, İhsan Kıvanç Gürsoy, Merve Hilal Dönmez, Juliette Fitremann, Arslan Bayram, Keziban Korkmaz Bayram

**Affiliations:** 1 Department of Translational Medicine, Institute of Health Sciences, Ankara Yıldırım Beyazit University, Ankara, Türkiye; 2 Department of Medical Genetics, Faculty of Medicine, Ankara Yıldırım Beyazit University, Ankara, Türkiye; 3 Faculty of Process Sciences, Institute of Biotechnology, Technical University of Berlin, Berlin, Germany; 4 SOFTMAT, Université de Toulouse, CNRS UMR 5623, Toulouse, France; 5 GENTAN, Genetic Diseases Evaluation Center, İzmir, Türkiye; 6 Izmir Biomedicine and Genome Center, İzmir, Türkiye

**Keywords:** Apoptosis, Cell survival, Maternal deprivation, 3D hippocampal cell culture, Nucleofection

## Abstract

Early-life stress (ELS), such as maternal separation, has been associated with neuronal apoptosis and impaired hippocampal function in rodent models. This study investigated the long-term effects of two ELS paradigms—unpredictable maternal separation (MS) and MS combined with unpredictable maternal stress (MSUS)—on the hippocampal mRNA expression of proapoptotic (*Bax*,* Tp53* and *Casp3*) and prosurvival (*Bcl2*) genes in offspring, both in vivo and in a complementary in vitro neuronal culture model. The study included three groups (Control, MS and MSUS groups), with ELS applied from postnatal days 1 to 14 to *BALB/c* mice. For the in vitro experiments, total RNA from the MSUS hippocampus was nucleofected at different concentrations into healthy mouse hippocampal cells, followed by 3D neuronal cell culture using N-heptyl-D-galactonamide (GalC7) hydrogels. Quantitative real-time PCR was used to assess gene expression, which was analyzed via the comparative Ct method (2^−ΔΔCt^). This study revealed that *Bax* mRNA expression was significantly lower in the MS group than in the control group, whereas both the MS and MSUS groups presented significant increases in *Bcl2* mRNA expression. In addition, the expression ratio of *Bcl2/Bax* was significantly greater in the MS and MSUS groups. No significant differences in *Tp53* or *Casp3* mRNA expression levels were detected between the groups. Although the in vitro mRNA expression levels were not significantly different, the mRNA expression ratio of *Bcl2/Bax* reached equilibrium as the concentration of total RNA nucleofected increased. Our results suggested that, in response to ELS, hippocampal cells adapt to prioritize survival over apoptosis.

**Supplementary Information:**

The online version contains supplementary material available at 10.1007/s11064-026-04822-7.

## Introduction

The early postnatal period is a critical time for neurodevelopment, particularly in the hippocampus. During this window, environmental factors can significantly impact brain development and potentially lead to long-lasting alterations in neural circuitry and behavior. The hippocampus, a brain region critical for memory formation [1, 2], is especially vulnerable to stress during early development. Stress can impair memory performance, alter synaptic plasticity and neuronal firing patterns, and induce structural modifications in the hippocampus [3].

One of the most widely used models to study early-life stress (ELS) in animals is maternal separation (MS). ELS, such as MS, has been associated with an increased risk for various neuropsychiatric disorders later in life [4–6]. Excessive exposure to glucocorticoids due to stress has been associated with hippocampal dysfunction and neuronal loss [7]. Moreover, early-life challenges that begin as early as pregnancy can have long-term effects by influencing neuronal apoptosis in the hippocampus, which in turn contributes to learning deficiencies in offspring [8].

In rodents, ELS and repetitive 3-hour MS have been shown to impact hippocampal function, particularly in relation to apoptosis and neuronal proliferation of offspring. For example, MS during postnatal days (PNDs) 2 to 14 has been linked to a reduction in mature neurons in the CA3 region of the hippocampus, and affects cognitive functions and maternal behavior in adult female mice [9]. It has also been associated with the epigenetic upregulation of corticotropin-releasing hormone, which contributes to synaptic dysfunction and memory impairments in adult rats [10]. Interestingly, early maternal and social deprivation during PND1–14 has also been reported to expand neural stem cell populations, increase neurogenesis in the hippocampus and amygdala, and reduce fear memory in mice [11]. Research in animal models consistently demonstrates that early-life stressors, such as MS, have detrimental effects on behaviors in adulthood [12, 13].

Increased apoptosis of pyramidal neurons in the dentate gyrus (DG) region has been reported in 3-week-old rats with a history of MS (PND3–21) [14]. Increased apoptosis in the DG has also been reported in 4-week-old rats subjected to 6-hour MS from PND1–14 [15]. Additionally, a significant reduction in the number of neurons in the DG has been reported in adult mice previously exposed to a single 24-hour MS on PND9, possibly due to reduced neurogenesis or increased neuronal apoptosis [16].

The effects of MS are thought to involve both the pups and the dam, including changes in maternal behavior and physiological parameters such as elevated stress hormones in the dam’s milk [13]. To investigate whether additional maternal stress exacerbates these effects and contributes to alterations in hippocampal apoptosis, we included the maternal separation with unpredictable stress (MSUS) paradigm, in which the dam is exposed to random stressors during separation [13]. Previous findings indicate that MSUS intensifies behavioral alterations in offspring when adult, suggesting that increased maternal stress can amplify the impact of ELS [13]. Importantly, these effects have been shown to be independent of maternal care or other environmental factors, implying a potential nongenomic mode of transmission [13].

Despite the well–known long–term impact of early MS and MSUS, their impacts on hippocampal apoptosis are not known, especially when MS is applied during PND1–14. This period in rodents is particularly critical, as it coincides with intense postnatal brain maturation [17] and overlaps with what is known as the stress-hyporesponsive period (SHRP). SHRP is characterized by a physiological state in which the HPA axis exhibits reduced sensitivity to stressors, although stress-responsive gene expression in the brain remains inducible [18]. Moreover, most previous studies have examined the outcomes of early MS in adulthood, whereas few have addressed the intermediate effects prior to reproductive maturity. To address this gap, we investigated the expression of four genes involved in regulating apoptosis: *Bax*,* Bcl2*,* Casp3*, and *Tp53*, in early mature adult mice (5 weeks old) [19]. These genes play crucial roles in determining cell fate during neurodevelopment and in response to stress. *Bax* promotes apoptosis [20–22], whereas *Bcl2* inhibits it [23]. *Casp3* is a key executioner caspase in the apoptotic cascade [24], and *Tp53* is a tumor suppressor gene that can induce apoptosis in response to cellular stress [25].

Using quantitative real-time PCR (qPCR), we analyzed the mRNA expression levels of these genes in the hippocampi of the mice at PND35, three weeks after the cessation of the stress paradigms. This time point allows us to assess the persistent effects of ELS on gene expression beyond the immediate stress response period.

Additionally, we employed nucleofection procedures to explore how different doses of total RNA from MSUS affect apoptosis-related gene expression in healthy hippocampal cells. The rationale for nucleofecting RNA into healthy neurons, rather than directly isolating MSUS-affected cells, was to determine whether RNA molecules alone are sufficient to initiate molecular reprogramming in a naïve environment. This approach serves as a mechanistic probe to test the capacity of RNA as a vehicle for non-Mendelian inheritance, providing a proof-of-concept for the role of hippocampal RNA cargo in transmitting stress-induced apoptotic signals. To observe these potential molecular shifts within a more physiologically relevant microenvironment that mimics tissue architecture, we cultured these cells on GalC7 hydrogels as a 3D cellular model. The GalC7 hydrogel was selected because it is composed of a single, pure molecule, which means that its composition is highly reproducible. The gels formed have low moduli, on the order of a few kPa, making them suitable for neuronal cell culture. Finally, the gel architecture is made up of quite wide and sparse fibers, which enable the cells to grow partly embedded within the gel. The degree of cell adhesion is also low, encouraging cells to organize themselves in small clusters along the fibers, thus reproducing a 3D culture quite well [26, 27].

Previous studies have reported the microinjection of RNA to create mouse models for autism [28, 29]. Similarly, our approach involves nucleofecting RNA from the hippocampi of stressed mice into healthy cells to test whether the stress-altered RNA content alone is sufficient to induce downstream apoptotic gene responses in vitro. Rather than aiming to replicate the full MSUS phenotype, this method allows us to isolate and assess the potential molecular impact of ELS-altered RNA in a controlled, cellular model, providing further insights into the molecular mechanisms underlying stress-related disorders.

## Materials and Methods

### Animals

This study was conducted in accordance with the institutional guidelines for the care and use of laboratory animals. Ethical clearance was obtained from Ankara University Laboratory Animals and Research Laboratory (Ethics Committee Approval No: 2023-3-20). Eight-week-old female *BALB/c* mice (*n* = 6 per group) were used to generate offspring. All animals were housed under a 12:12 h light/dark cycle (lights at 7 a.m. to 7 p.m.) with constant room temperature (22 ± 2 °C) and humidity (45 ± 5%) in standard plastic mouse cages (22 cm × 38 cm × 15 cm). Food and tap water were available *ad libitum*. All the cages were subjected to weekly cage cleaning. The breeding procedures included housing one female and one male in the same cage. After the mating period, the males were removed, and the female mice remained alone throughout the gestation period.

### Maternal Stress Procedures

This study included three groups of litter from related dams: control, unpredictable maternal separation (MS), and unpredictable maternal separation combined with unpredictable maternal stress (MSUS) groups. The maternal stress model was re-established following the same procedures described in our previous study [30]. Briefly, dams and litters were separated for 3 h each day from postnatal day (PND) 1 to PND 14. Additionally, MSUS dams experienced unpredictable maternal stress in conjunction with the MS procedure [13, 31], consisting of a forced swim test (6 min) or restraint stress (20 min) applied interchangeably during the final minutes of the 3-hour MS daily from PND1 to PND14. A total of 72 five-week-old litters, with 24 (12 female litters and 12 male litters) from each group, were used in the in vivo study. Additionally, five-week-old control male litters (*n* = 4) were used for the in vitro 3D hippocampal cell culture experiments.

### Tissue Collection and Hippocampal Dissociation into Single Cells

At PND35, the mice were sacrificed by cervical dislocation. The hippocampi were dissected and either stored at − 80 °C for RNA isolation or immediately processed for in vitro 3D hippocampal culture. Hippocampal dissociation into single cells was performed via enzymatic digestion with trypsin, followed by washing, resuspension in prewarmed culture medium, and mechanical trituration to obtain a homogeneous cell suspension. The detailed steps and solution compositions are provided in the Supplementary Methods.

### Cell Counting and Viability Assessment

An additional 5 mL of culture medium was added to the hippocampal cells, and the cells were counted using a TC20 cell counter (Bio-Rad, USA). The average hippocampal cell count was 5.91 × 10⁶ cells/mL. The counted cells were centrifuged at 80 × *g* for 10 min, and the supernatant was discarded. Cell viability was assessed under a microscope using 0.4% trypan blue solution (Gibco, Life Technologies) at a 1:1 ratio. Once cell viability was confirmed to be suitable, nucleofection was performed.

### Total RNA Isolation from Hippocampal Tissue

The collected hippocampal tissues were transferred into Eppendorf tubes containing 500 mL of NucleoGene Tri Reagent Lysis Reactive (NucleoGene, Türkiye). The tissues were disintegrated via an ultrasonic tissue homogenizer. Total RNA was isolated from hippocampal tissue (*n* = 72) via the phenol‒chloroform extraction method as described in a previous study [32]. The total RNA used for nucleofection was selected on the basis of the criteria of increased *Bcl2* and decreased *Bax* expression, and was obtained from the hippocampus of a male litter in the MSUS group. Male mice were used to avoid the potential confounding effects of hormonal fluctuations associated with the estrous cycle in female mice. The total RNA was further purified via the High Pure RNA Isolation Kit (Roche, Germany) to eliminate any DNA-induced effects. The purity and concentration of the total RNA were measured with a NanoDrop 2000c (Thermo Fisher Scientific, USA).

### Preparation of the Hydrogel

The GalC7 molecule was synthesized as previously described [26], and the GalC7 hydrogel was prepared for culture as described in our previous study [32] Briefly, GalC7 powder was dissolved in nuclease-free water at 115 °C and then transferred to culture plate wells for gelation. The gels were saturated with culture medium and incubated at 37 °C with 5% CO₂ before use. The complete preparation steps are described in the Supplementary Methods.

### Nucleofection and 3D Cell Culture using the GalC7 Hydrogel

Exogenous RNA (total RNA, RNAse-treated total RNA (mock) and 0.5, 1, and 2 ng/µL diluted from total RNA) was nucleofected into control male hippocampal cells via the Amaxa^®^ 4D-Nucleofector^®^ Protocol for Primary Mammalian Neurons (Lonza, Switzerland), following the previously optimized DR-114 program, as indicated in the kit protocol. After nucleofection, the nucleocuvette was incubated for 10 min at room temperature. After incubation, the cells were resuspended in prewarmed medium and gently mixed by pipetting up and down 2–3 times. The nucleofected cells were cultured with GalC7 gel prepared in a 48-well plate. A total of 250 µL of DMEM containing 10% penicillin/streptomycin (Gibco, Life Technologies), and 1X B27 supplement (Gibco, Life Technologies) were added. The samples were incubated at 37 °C with 5% CO2 for seven days. Four samples from each group were prepared: a control group without nucleofection; a mock-nucleofected group; 0.5, 1 and 2 ng/µL total RNA-nucleofected groups. These samples were cultured in GalC7 for a 7-day culture period. After 7 days, the culture was terminated and RNA isolation was initiated. The medium was removed, and the samples were washed with PBS. The samples were lysed with 1 mL of NucleoGene Tri Reagent Lysis Reactive. Total RNA was extracted from 3D hippocampal cell cultures via a phenol-chloroform method that was previously established [32].

### cDNA Synthesis and Quantitative Real-Time PCR

cDNA synthesis from total RNA was performed with the HiScript II 1st Strand cDNA Synthesis Kit (Vazyme, China). Each sample was adjusted to approximately 1 µg of total RNA. The reaction mixture for each sample consisted of 10 µL of 2x RT Mix, 2 µL of HiScript II Enzyme Mix, 1 µL of Oligo-(dT)23 VN (50 µM), 1 µL of random hexamers (50 ng/µL), 4 µL of total RNA, and 2 µL of nuclease-free water, yielding a total reaction volume of 20 µL. cDNA synthesis was carried out via T100 Thermal Cycler PCR (Bio-Rad, USA) with the following profile: 5 min at 25 °C, 15 min at 50 °C, and 2 min at 85 °C. The resulting cDNA samples were diluted 1:5 with nuclease-free water.

Quantitative real-time PCR was performed on a Rotor-Gene Q (Qiagen, Germany) with specific primers (Table 1) and 2x ChamQ Universal SYBR qPCR Master Mix (Vazyme, China). The reaction mixture consisted of 10 µL of 2x ChamQ Universal SYBR qPCR Master Mix, 0.8 µL of 10 µM forward and reverse primer mixture, 6.2 µL of nuclease-free water, and 3 µL of 1:5 diluted cDNA, totaling 20 µL per qPCR.

The qPCR profile was as follows: 30 s at 95 °C for initial denaturation, 10 s at 95 °C for denaturation, and 30 s at 58 °C for annealing and extension, followed by melting curve analysis. The cycle count was set to 45. Each sample was repeated at least twice. The results were analyzed via the delta‒delta‒Ct (2^–ΔΔCt^) method. The data were normalized to the reference gene *Gapdh*, and the mean values from the control group were used as the calibrator.

**Table 1:** Sequences of the primers

Primer	Sequence (5’-3’)	Amplicon length (bp)
*Gapdh*	Forward: CTCTCTGCTCCTCCCTGTTC Reverse: TACGGCCAAATCCGTTCACA	105
*Bax*	Forward: TTTGCTACAGGGTTTCATCCA Reverse: ATATTGCTGTCCAGTTCATCTCC	147
*Bcl2*	Forward: CTGGGATGCCTTTGTGGAAC Reverse: TCAAACAGAGGTCGCATGCT	51
*Casp3*	Forward: CAGCACCTGGTTACTATTCCTG Reverse: TTCCTGTTAACGCGAGTGAG	130
*Tp53*	Forward: CAACAGCTCCTGCATGGGGGGC Reverse: AGGACAGGCACAAACACGAACC	121

### Statistical Analyses

Statistical analyses were conducted via GraphPad Prism 9.1.0 (GraphPad Software, USA). The distribution of the data was assessed through histograms, q-q plots, and Shapiro‒Wilk tests. The outliers were identified and removed via the ROUT method in GraphPad Prism (Q = 1%). Independent t tests or Mann‒Whitney U tests were used to compare data between two groups. For calculation of the *Bcl2/Bax* ratio, the relative quantification units of *Bcl2* were divided by those of *Bax* for each sample. For comparisons involving three or more groups, the Kruskal‒Wallis H test was employed to assess the significance of the difference in accordance with the distribution of the data. Dunn’s test was used for post hoc analysis. The relationships between the data were evaluated via Spearman correlation. Statistical significance was considered at a *P* value of < 0.05.

## Results

**Fig. 1:**
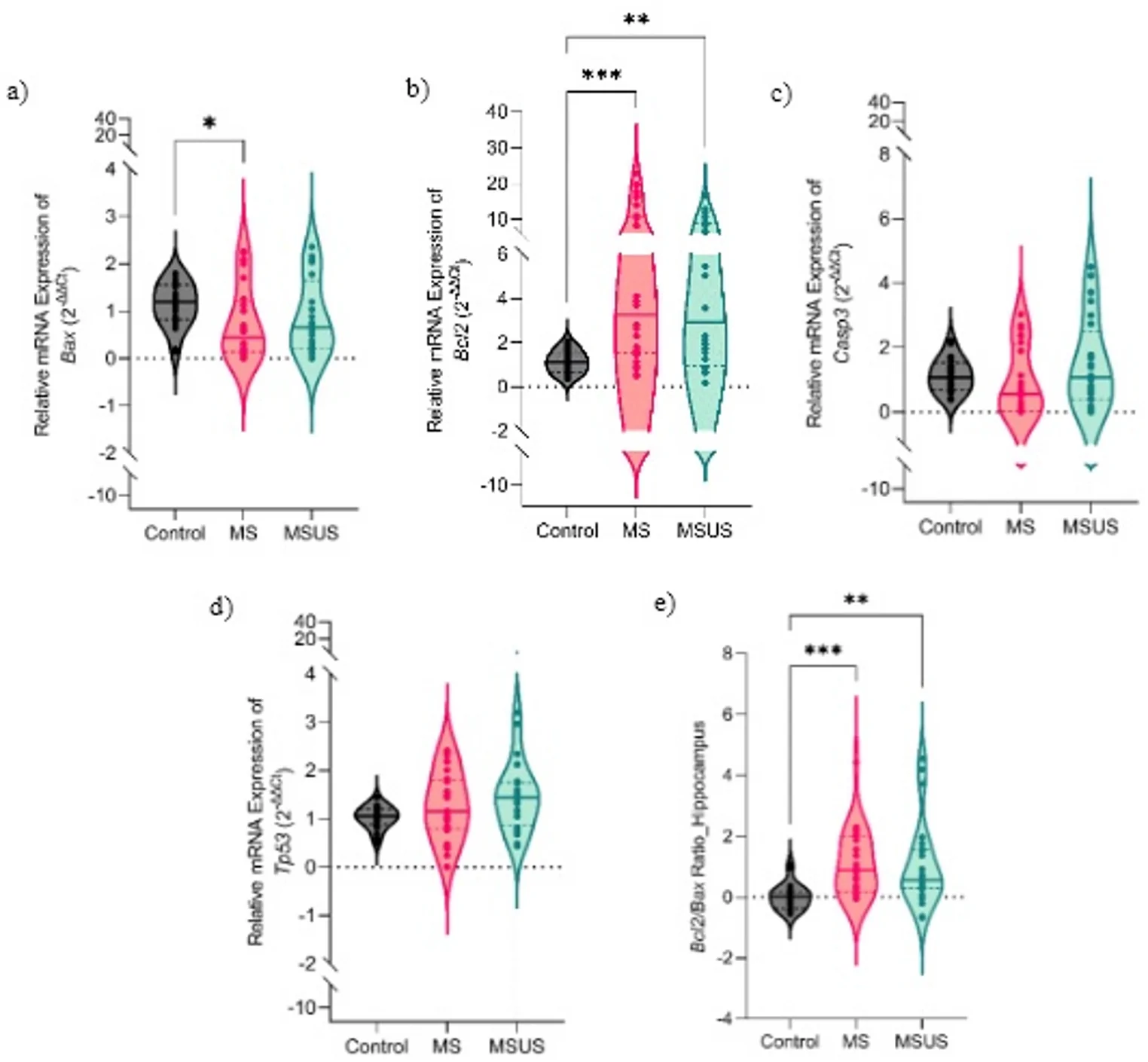
Violin plots showing the relative mRNA expression of *Bax* (**a**), *Bcl2* (**b**), *Casp3* (**c**), and *Tp53* (**d**) and the *Bcl2/Bax* expression ratio (**e**) in the hippocampus of litters subjected to early-life maternal separation stress. The y-axis represents relative mRNA expression, and the x-axis represents the control (*n* = 24), MS (*n* = 24), and MSUS (*n* = 24) groups. Each plot includes individual data points, the first quartile (Q1), the third quartile (Q3), and the median. The descriptive statistics (mean ± SD, median, Q1, and Q3) for each gene are as follows: *Bax*: control (1.12 ± 0.46, 1.20, 0.82, and 1.55), MS (0.74 ± 0.77, 0.45, 0.14, and 1.22), and MSUS (0.86 ± 0.79, 0.64, 0.20, and 1.63); *Bcl2*: control (1.13 ± 0.49, 1.11, 0.64, and 1.50), MS (6.58 ± 6.86, 3.26, 1.50, and 10.85), and MSUS (4.94 ± 4.64, 2.92, 0.96, and 8.75); *Casp3*: control (1.12 ± 0.52, 1.06, 0.67, and 1.52), MS (0.95 ± 1.07, 0.55, 0.04, and 2.09), and MSUS (1.49 ± 1.40, 1.06, 0.40, and 2.49); *Tp53*: control (1.03 ± 0.23, 1.06, 0.89, and 1.20), MS (1.27 ± 0.70, 1.16, 0.79, and 1.81), and MSUS (1.45 ± 0.70, 1.44, 0.88, and 1.75); and *Bcl2/Bax*: control (0.01 ± 0.41, 0.02, -0.07, and 0.19), MS (1.11 ± 1.10, 0.89, 0.16, and 1.98), and MSUS (1.03 ± 1.39, 0.56, 0.30, and 1.56)

The mRNA expression levels of *Bax*,* Bcl2*,* Casp3*, and *Tp53* in hippocampal tissue are presented in Fig. 1a-d. Prior to conducting the main analysis, including the Kruskal‒Wallis test, an independent t test was performed to confirm the absence of significant differences between male and female offspring across all genes and experimental groups (*P* > 0.05), allowing us to combine the data. Given that the data did not meet the assumption of a normal distribution, a nonparametric Kruskal‒Wallis test was performed. Our study revealed significant differences in the mean ranks of *Bax* mRNA expression among the groups (*H* (2, *n* = 72) = 6.38, *P =* 0.04; Fig. 1a). Dunn’s multiple comparison test revealed significant downregulation in the MS group compared with the control group (*P* = 0.05). For *Bcl2*, significant differences in mean ranks were observed (*H* (2, *n* = 72) = 16.22, *P =* 0.0003; Fig. 1b). Dunn’s multiple comparison test revealed significant upregulation in both the MS and MSUS groups compared with the control group (*P* = 0.0005 and *P* = 0.0057, respectively). No significant differences were detected among the groups for either *Casp3* (*H* (2, *n* = 72) = 3.43, *P >* 0.05; Fig. 1c) or *Tp53* (*H* (2, *n* = 72) = 5.50, *P >* 0.05; Fig. 1d). Furthermore, our study revealed significant differences in the mean ranks of the *Bcl2/Bax* expression ratios among the groups (H (2, *n* = 72) = 20.16, *P* < 0.0001; Fig. 1e). Dunn’s multiple comparison test revealed a significant increase in the *Bcl2/Bax* expression ratio in both the MS and MSUS groups compared with the control group (*P* = 0.0001 and *P* = 0.0010, respectively).

**Table 2:** Spearman correlation coefficients between Bax, Bcl2, Casp3 and Tp53 mRNA expression in the hippocampus of litters within each experimental group

		1. *Bax*	2. *Bcl2*	3. *Casp3*	4. *Tp53*
Control	**1.**	1			
	**2.**	-0.40	1		
	**3.**	0.24	0.01	1	
	**4.**	0.61**	-0.43*	0.21	1
MS	**1.**	1			
	**2.**	-0.18	1		
	**3.**	0.61**	-0.10	1	
	**4.**	0.74****	-0.11	0.37	1
MSUS	**1.**	1			
	**2.**	0.07	1		
	**3.**	0.68***	0.23	1	
	**4.**	0.65***	0.04	0.58**	1

*p < 0.05

**p < 0.01

***p < 0.001

****p < 0.0001

Spearman’s rank correlation was performed to further explore the relationships between gene expression in the hippocampal tissue of the litters. As shown in Table 2, in the control group, a significant positive correlation was observed between *Bax* and *Tp53* expression (*r* = 0.61, *P* = 0.002), whereas *Bcl2* and *Tp53* expression was significantly negatively correlated (*r*=-0.43, *P* = 0.034). In the MS group, significant positive correlations were observed between *Bax* and *Tp53* (*r* = 0.74, *P <* 0.0001) and between *Bax* and *Casp3* (*r* = 0.61, *P* = 0.0015). In the MSUS group, significant positive correlations were detected between *Bax* and *Tp53* (*r* = 0.65, *P =* 0.0006), between *Bax* and *Casp3* (*r* = 0.68, *P* = 0.0003), and between *Casp3* and *Tp53* (*r* = 0.58, *P* = 0.0027).

**Fig. 2:**
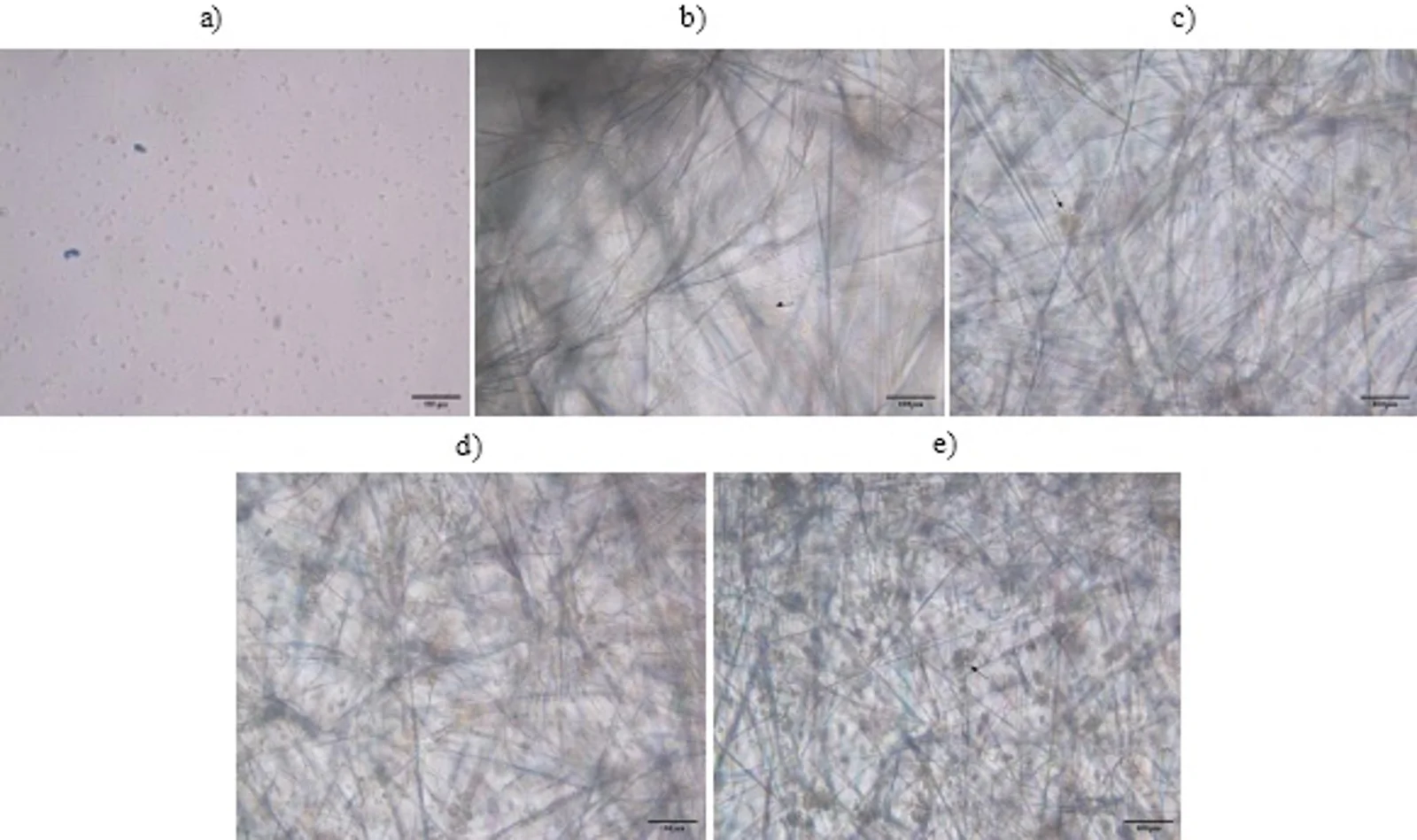
Viability and development of hippocampal cells in 3D GalC7 hydrogels. **a** Trypan blue staining revealed mostly viable cells postnucleofection. **b**-**e** Hippocampal cells within the GalC7 hydrogels on Day 1 (**b**), Day 3 (**c**), Day 5 (**d**), and Day 7 (**e**), as captured via an Olympus CKX41 inverted microscope at 20x magnification (scale bars: 100 μm)

Trypan blue staining confirmed that most cells remained viable postnucleofection (Fig. 2a). Additionally, the development of hippocampal cells in GalC7 was monitored over time via an Olympus CKX41 inverted microscope at 20x magnification (Fig. 2b-e). Initially, the cells appeared sparsely distributed with minimal neurite outgrowth. Over time, increased cell clustering was observed, along with more prominent interactions between cells and the surrounding GalC7 fibers. In later stages, the cells appeared more densely distributed and organized within the hydrogel, suggesting progressive maturation within the 3D environment.

**Fig. 3:**
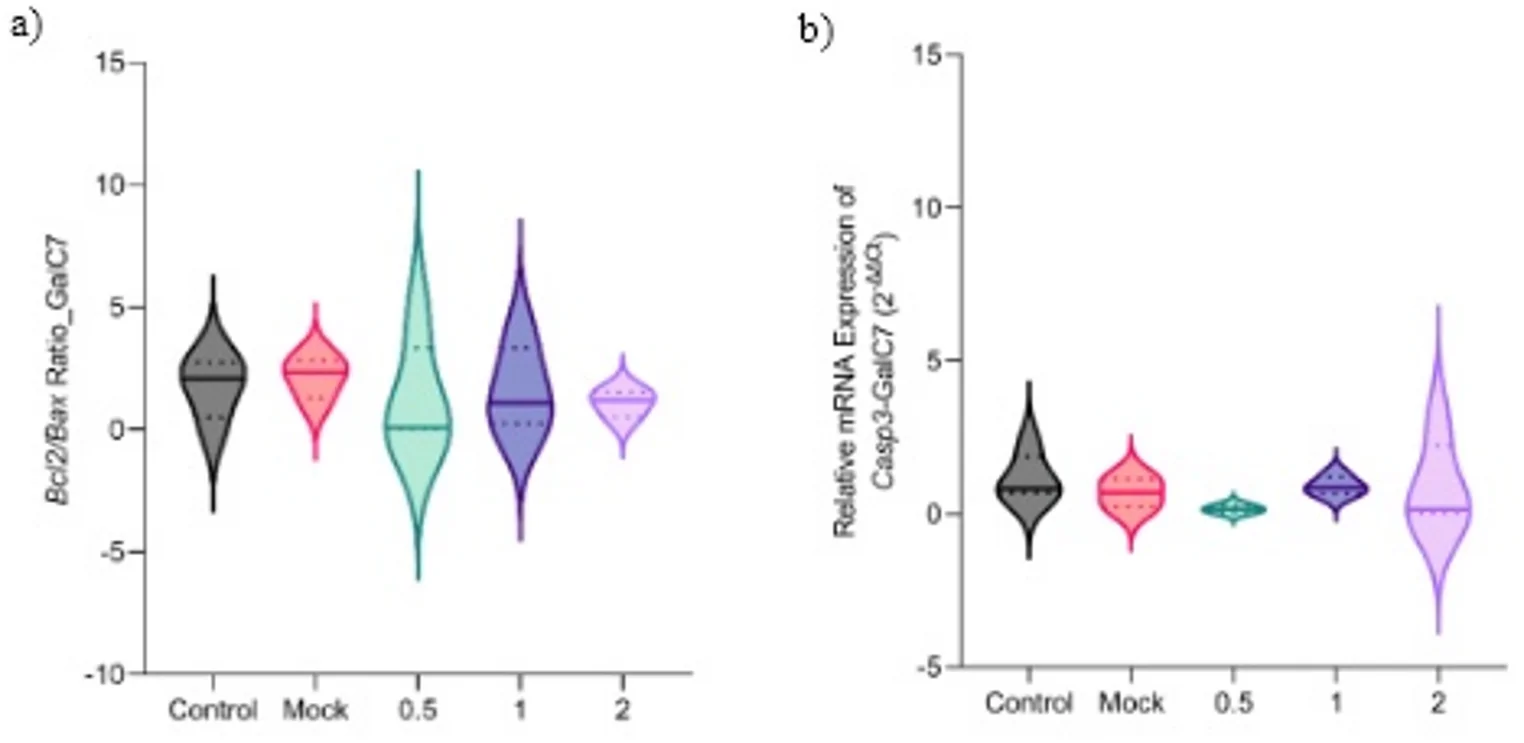
Violin plot showing the *Bcl2/Bax* expression ratio in 3D cultured hippocampal cells (**a**) and the relative mRNA expression of *Casp3* (**b**) in 3D cultured hippocampal cells. The y-axis represents relative mRNA expression, and the x-axis represents the control (*n* = 4), mock (*n* = 4), 0.5 (*n* = 4), 1 (*n* = 4), and 2 (*n* = 4) groups. Each plot includes individual data points, the first quartile (Q1), the third quartile (Q3), and the median. The descriptive statistics (mean ± SD, median, Q1, and Q3) for each group are as follows: *Bcl2/Bax*: control (1.78 ± 1.21, 2.09, 0.51, and 2.74), mock (2.16 ± 0.81, 2.34, 1.31, and 2.82), 0.5 (1.15 ± 2.18, 0.08, 0.04, and 3.34), 1 (1.57 ± 1.69, 1.10, 0.25, and 3.34), and 2 (1.09 ± 0.54, 1.21, 0.52, and 1.54); *Casp3*: control (1.14 ± 0.74, 0.83, 0.69, and 1.89), mock (0.69 ± 0.49, 0.70, 0.23, and 1.14), 0.5 (0.17 ± 0.13, 0.16, 0.05, and 0.30), 1 (0.91 ± 0.30, 0.86, 0.66, and 1.20), and 2 (0.81 ± 1.39, 0.16, 0.04, and 2.23)

The *Bcl2/Bax* expression ratios in 3D hippocampal cell culture samples from different groups (control, mock, 0.5, 1, and 2) are presented in Fig. 3a. No significant differences were observed across groups (H (4, *n* = 20) = 3.77, *P* > 0.05). Notably, the *Bcl2/Bax* ratios in the control and mock groups were similar, which is important for ruling out potential effects of nucleofection on gene expression. Although no significant differences were found, the *Bcl2/Bax* ratio was lower in the nucleofected groups than in the control and mock groups. The highest variability in the *Bcl2/Bax* ratio was observed in the 0.5 group, suggesting a heterogeneous response at this concentration, whereas the variability decreased at higher concentrations.

Furthermore, the mRNA expression levels of *Casp3* in 3D hippocampal cell culture samples from different groups (control, mock, 0.5, 1, and 2) are presented in Fig. 3b. No significant differences were observed across groups for *Casp3* (*H* (4, *n* = 20) = 7.91, *P* > 0.05).

## Discussion

In this study, we investigated the long-term effects of PND1-14 postnatal unpredictable maternal separation (MS) and unpredictable maternal separation combined with unpredictable maternal stress (MSUS) on apoptosis-related gene expression in the hippocampus of five-week-old mice. While several studies have reported that MS leads to increased apoptotic cell death in the hippocampi of rats and mice, notably at various time intervals [14–16, 33], our findings revealed that survival tends to be promoted instead of apoptotic pathways in the hippocampus. In our study, the expression of the *Bcl2* mRNA was significantly greater in the MS and MSUS groups than in the control group (*P* = 0.0005 and *P* = 0.0057, respectively). Compared with that in the control group, *Bax* was significantly downregulated in the MS group (*P* = 0.05) but not in the MSUS group. However, MSUS *Bax* expression still consistently decreased, similar to MS. In terms of their interaction, the control correlation results, *Bax* and *Bcl2* showed a nonsignificant moderate negative correlation (*r*= -0.40), reflecting their classical antagonistic role in regulating apoptosis. However, this correlation weakened in MS (*r*= -0.18) and disappeared in MSUS (*r* = 0.07), suggesting that their regulatory relationship is disrupted under MS and MSUS conditions. The upregulation of *Bcl2* in MS and MSUS indicates the promotion of hippocampal neuronal survival while simultaneously suppressing apoptotic processes. Additionally, the significantly increased *Bcl2/Bax* ratios in the MS (*P* = 0.0001) and MSUS (*P* = 0.0010) groups compared with those in the control group further support a shift toward cell survival mechanisms.

Previous studies have shown that ELS, such as low-level maternal care, increases vulnerability to hippocampal neuron loss via apoptosis in PND90 rats [34]. In humans, early-life adversity has been linked to a smaller hippocampus volume in male depressed in-patients [35]; this alteration may stem from the apoptotic pathway and contribute to severe neuropsychiatric outcomes. Moreover, early MS has been associated with long-term consequences [36]. Our findings suggest that hippocampal cells adapt to promote survival, counteract apoptosis and preserve neuronal integrity. This response may help mitigate the long-term impact of ELS and prevent more severe consequences later in life. Neuronal survival can be initiated through increased intracellular Ca²⁺, which activates prosurvival signal transduction pathways (the phosphoinositide 3-kinase (PI3K)/Akt pathway, the protein kinase C (PKC)/extracellular signal-regulated kinase (ERK) pathway [14], and the Ca^2+^/calmodulin pathway), which further inhibits the transcription of cell death-related genes and activates key transcription factors such as CREB and NF-κB. This leads to an increase in *Bcl2* expression [37, 38] and an increase in the ratio of antiapoptotic to proapoptotic factors within the cell [39], possibly due to an increase in hippocampal IL-1β and TNF-α via those pathways, as reported in some short- and long-term maternal separation studies [40]. In support of this possible mechanism, our preliminary data suggest a decrease in the expression of Ca²⁺-impermeable *Gria2* in MS and MSUS. This may imply enhanced Ca²⁺ influx, which could be involved in the upregulation of *Bcl2*, possibly supporting neuronal survival. The elevated *Bcl2* mRNA expression observed in our study, which focused on the prepubertal period, may possibly persist into adulthood, as suggested by the study of Coccurello. et al. [41], who reported increased *Bcl2* mRNA expression in the hippocampus of mice at PND90 following early-life separation [41].

There were no significant differences in *Casp3* expression among the groups. There was variability in expression, but there was no strong trend toward upregulation or downregulation. This result is consistent with the notion that the transcription of the prosurvival protein *Bcl2* and the repression of the proapoptotic protein *Bax* likely prevent the activation of the subsequent caspase cascade, including the Casp3-dependent apoptotic cascade. Despite the lack of significant differences, *Casp3* maintained strong positive correlations with *Bax* under MS (*r* = 0.61) and MSUS (*r* = 0.68) conditions. *Tp53* expression did not significantly change; however, its significant strong correlation with *Bax* expression in MS (*r* = 0.74) and MSUS (*r* = 0.65) suggests that while survival conditions are maintained, there may not be complete suppression of apoptosis.

In addition to our in vivo study, we evaluated how different RNA doses (0.5, 1, and 2 ng/µL) of MSUS affect apoptosis-related gene expression in healthy hippocampal cells cultured on GalC7 hydrogels. Although statistical significance was not reached, several key observations provide insights into the potential of this in vitro model. The similarity in gene expression between the control and mock-nucleofected groups indicates that the nucleofection process itself does not induce apoptosis-related changes. Moreover, in the 3D hippocampal cell culture nucleofected with 0.5 ng/µL total RNA, the *Bcl2/Bax* ratio was lower than that in the control and mock-nucleofected groups; however, this decrease was not statistically significant. As the nucleofected total RNA concentration increased, the ratio approached the levels observed in the control and mock groups, with notably reduced variability. Neuronal cells, which are particularly challenging to culture in 3D, are expected to undergo prominent apoptosis. The elevated *Bcl2* expression observed in the hippocampus of stressed mice may contribute to promoting a survival-supportive transcriptome. Whereas our in vitro data do not significantly demonstrate improved survival, the observed stabilization of the *Bcl2/Bax* ratio with increasing RNA concentration suggests that nucleofection of stress-derived RNA may partially recapitulate adaptive transcriptomic signatures. These in vitro findings underscore the potential importance of RNA concentration in modulating apoptosis-related gene expression in ELS models.

Despite the limitations of this study, particularly the lack of detailed characterization of the total RNA extracted from the hippocampus of male mice in the MSUS group used for nucleofection, we confirmed that the RNA sample used for nucleofection was extracted from tissue exhibiting an in vivo expression profile of increased *Bcl2* and decreased *Bax* levels, whereas the mRNA expression levels of *Casp3* and *Tp53* did not show comparable expression to those in the control group. Another limitation of this study is that neuronal markers were not assessed in 3D hippocampal culture. However, previous work with GalC7 hydrogels [26, 32] demonstrates that it provides a biomimetic scaffold that supports high-density cell clustering and neuronal maturation. Furthermore, this study is limited to mRNA transcript analysis, which does not necessarily reflect corresponding protein levels or biological activity. Therefore, future studies incorporating protein-level validation will be essential to confirm the functional relevance of the observed transcriptomic changes. While this study focuses on the early molecular precursors of apoptosis, we acknowledge that the absence of concurrent histological data (such as TUNEL assay) at this specific point is another limitation. Future studies involving long-term follow-ups and functional assays will be essential to determine how these early molecular adaptations translate into long-term phenotypic outcomes.

## Conclusion

We concluded that unpredictable early-life MS and MSUS stress significantly affect the mRNA expression of apoptosis-related genes in the hippocampus of five-week-old *BALB/c* mice. Specifically, the mRNA expression of prosurvival genes increased, whereas the mRNA expression of proapoptotic genes decreased. These expression patterns were similar in both sexes, suggesting the involvement of non–sex-dependent mechanisms. Additionally, the presence of extra maternal stress in the MSUS paradigm did not significantly alter the gene expression profile compared with that in the MS-only condition. The alterations observed may be an adaptive mechanism supporting neuronal survival in adolescents. If early-life maternal deprivation or MS leads to widespread apoptosis in the hippocampus — a region where neurogenesis continues into adulthood — the detrimental effects are likely to manifest as an increased susceptibility to neurodegenerative diseases. However, the brain possesses adaptive mechanisms to mitigate severe neuronal loss. In this study, the activation of survival pathways in hippocampal cells may reflect an adaptive response, protecting against major stress-induced damage. These findings may inform future strategies for early intervention aimed at reducing the long-term neurobiological consequences of ELS.

## Supplementary Information

Below is the link to the electronic supplementary material.

Supplementary Material 1.

## Data Availability

The datasets generated and/or analyzed during the current study are available from the corresponding author upon reasonable request.

## References

[1] Hammoud K, Spittler N, Buch K et al (2019) Camping Out in the Hippocampus: Imaging Spectrum of Etiologies That Affect the Hippocampi and Tips for Honing the Differential Diagnosis. Neurographics 9:37–54. 10.3174/ng.1800032

[2] Costa V, Lugert S, Jagasia R (2015) Role of adult hippocampal neurogenesis in cognition in physiology and disease. Handb Exp Pharmacol. 228:99-155 10.1007/978-3-319-16522-6_4 25977081

[3] Kim EJ, Pellman B, Kim JJ (2015) Stress effects on the hippocampus: A critical review. Learn Mem 22:411–416. 10.1101/lm.037291.114 26286651 10.1101/lm.037291.114 PMC4561403

[4] Pagliaccio D, Barch DM (2016) Early Life Adversity and Risk for Depression: Alterations in Cortisol and Brain Structure and Function as Mediating Mechanisms. Elsevier Inc

[5] Wenderski W, Maze I (2014) Epigenetic Mechanisms of Drug Addiction Vulnerability. Elsevier Inc

[6] Pariante CM, Nemeroff CB (2012) Unipolar depression. Handb Clin Neurol 106:239–249. 10.1016/B978-0-444-52002-9.00014-0 22608625 10.1016/B978-0-444-52002-9.00014-0

[7] Lucassen PJ, Müller MB, Holsboer F et al (2001) Hippocampal Apoptosis in Major Depression Is a Minor Event and Absent from Subareas at Risk for Glucocorticoid Overexposure. Am J Pathol 158:453–468. 10.1016/S0002-9440(10)63988-0 11159183 10.1016/S0002-9440(10)63988-0 PMC1850286

[8] Kuang H, Sun M, Lv J et al (2014) Hippocampal apoptosis involved in learning deficits in the offspring exposed to maternal high sucrose diets. J Nutr Biochem 25:985–990. 10.1016/J.JNUTBIO.2014.04.012 24998948 10.1016/j.jnutbio.2014.04.012

[9] Reshetnikov VV, Kovner AV, Lepeshko AA et al (2020) Stress early in life leads to cognitive impairments, reduced numbers of CA3 neurons and altered maternal behavior in adult female mice. Genes Brain Behav 19:e12541. 10.1111/GBB.12541 30488555 10.1111/gbb.12541

[10] Wang A, Nie W, Li H et al (2014) Epigenetic Upregulation of Corticotrophin-Releasing Hormone Mediates Postnatal Maternal Separation-Induced Memory Deficiency. PLoS ONE 9:e94394. 10.1371/JOURNAL.PONE.0094394 24718660 10.1371/journal.pone.0094394 PMC3981802

[11] Daun KA, Fuchigami T, Koyama N et al (2020) Early Maternal and Social Deprivation Expands Neural Stem Cell Population Size and Reduces Hippocampus/Amygdala-Dependent Fear Memory. Front Neurosci 14:483850. 10.3389/FNINS.2020.00022/BIBTEX 10.3389/fnins.2020.00022 PMC7000530 32063832

[12] Gapp K, Soldado-Magraner S, Alvarez-Sánchez M et al (2014) Early life stress in fathers improves behavioural flexibility in their offspring. Nat Commun. 10.1038/ncomms6466 10.1038/ncomms6466 25405779

[13] Weiss IC, Franklin TB, Vizi S, Mansuy IM (2011) Inheritable effect of unpredictable maternal separation on behavioral responses in mice. Front Behav Neurosci. 10.3389/fnbeh.2011.00003 10.3389/fnbeh.2011.00003 PMC3034937 21331159

[14] Yang S, Li J, Han L, Zhu G (2017) Early maternal separation promotes apoptosis in dentate gyrus and alters neurological behaviors in adolescent rats. Int J Clin Exp Pathol 10:10812 31966424 PMC6965815

[15] Baek S, Bin, Bahn G, Moon SJ et al (2011) The phosphodiesterase type-5 inhibitor, tadalafil, improves depressive symptoms, ameliorates memory impairment, as well as suppresses apoptosis and enhances cell proliferation in the hippocampus of maternal-separated rat pups. Neurosci Lett 488:26–30. 10.1016/j.neulet.2010.10.074 21056623 10.1016/j.neulet.2010.10.074

[16] Fabricius K, Wörtwein G, Pakkenberg B (2008) The impact of maternal separation on adult mouse behaviour and on the total neuron number in the mouse hippocampus. Brain Struct Funct 212:403–416. 10.1007/s00429-007-0169-6 18200448 10.1007/s00429-007-0169-6 PMC2226080

[17] Kudryashov IE, Onufriev MV, Kudryashova IV, Gulyaeva NV (2001) Periods of postnatal maturation of hippocampus: synaptic modifications and neuronal disconnection. Dev Brain Res 132:113–120. 10.1016/S0165-3806(01)00301-7 11744115 10.1016/s0165-3806(01)00301-7

[18] Smith MA, Kim SY, Van Oers HJJ, Levine S (1997) Maternal deprivation and stress induce immediate early genes in the infant rat brain. Endocrinology 138:4622–4628. 10.1210/endo.138.11.5529 9348187 10.1210/endo.138.11.5529

[19] Radulescu CI, Cerar V, Haslehurst P et al (2021) The aging mouse brain: cognition, connectivity and calcium. Cell Calcium 94:102358. 10.1016/J.CECA.2021.102358 33517250 10.1016/j.ceca.2021.102358

[20] Brady HJM, Gil-Gómez G (1998) Molecules in focus bax. The pro-apoptotic Bcl-2 family member, bax. Int J Biochem Cell Biol 30:647–650. 10.1016/S1357-2725(98)00006-5 9695020 10.1016/s1357-2725(98)00006-5

[21] Dai Z, Lai JR (2019) Isolation of Synthetic Antibodies Against BCL-2-Associated X Protein (BAX). Methods Mol Biol 1877:351–357. 10.1007/978-1-4939-8861-7_21 30536015 10.1007/978-1-4939-8861-7_21 PMC7189900

[22] Wei MC, Zong WX, Cheng EHY et al (2001) Proapoptotic BAX and BAK: a requisite gateway to mitochondrial dysfunction and death. Science 292:727–730. 10.1126/SCIENCE.1059108 11326099 10.1126/science.1059108 PMC3049805

[23] Siddiqui WA, Ahad A, Ahsan H (2015) The mystery of BCL2 family: Bcl-2 proteins and apoptosis: an update. Arch Toxicol 89:289–317. 10.1007/S00204-014-1448-7 25618543 10.1007/s00204-014-1448-7

[24] Porter AG, Jänicke RU (1999) Emerging roles of caspase-3 in apoptosis. Cell Death Differ 6:99–104. 10.1038/SJ.CDD.4400476 10200555 10.1038/sj.cdd.4400476

[25] Pflaum J, Schlosser S, Müller M et al (2014) p53 Family and Cellular Stress Responses in Cancer. Front Oncol 4:285. 10.3389/FONC.2014.00285 25374842 10.3389/fonc.2014.00285 PMC4204435

[26] Chalard A, Vaysse L, Joseph P et al (2018) Simple Synthetic Molecular Hydrogels from Self-Assembling Alkylgalactonamides as Scaffold for 3D Neuronal Cell Growth. ACS Appl Mater Interfaces 10:17004–17017. 10.1021/acsami.8b01365 29757611 10.1021/acsami.8b01365

[27] Kasmi N, Pieruccioni L, Pitot E et al (2025) The potential of carbohydrate supramolecular hydrogels for long-term 3D culture of primary fibroblasts. J Mater Chem B. 10.1039/d4tb02658f 40084972 10.1039/d4tb02658f

[28] Ozkul Y, Taheri S, Bayram KK et al (2020) A heritable profile of six miRNAs in autistic patients and mouse models. Sci Rep. 10.1038/s41598-020-65847-8 10.1038/s41598-020-65847-8 PMC7280218 32514154

[29] Yilmaz Sukranli Z, Korkmaz Bayram K, Mehmetbeyoglu E et al (2024) Trans species RNA activity: sperm RNA of the father of an autistic child programs glial cells and behavioral disorders in mice. Biomolecules. 10.3390/BIOM14020201 10.3390/biom14020201 PMC10886764 38397438

[30] Bayram KK, Barokah AN, Dönmez MH et al (2024) Unravelling the maternal stress-induced orchestrations: Fndc5 gene expression dynamics across duodenum, stomach, and whole blood in offspring. Acta Med Cordoba 55:153–161. 10.32552/2024.ActaMedica.1003

[31] Gapp K, Corcoba A, van Steenwyk G et al (2017) Brain metabolic alterations in mice subjected to postnatal traumatic stress and in their offspring. J Cereb Blood Flow Metab 37:2423–2432. 10.1177/0271678X16667525 27604311 10.1177/0271678X16667525 PMC5531341

[32] Bayram KK, Fitremann J, Bayram A et al (2021) Gene expression of mouse hippocampal stem cells grown in a galactose-derived molecular gel compared to in vivo and neurospheres. Processes. 10.3390/pr9040716

[33] Lee HJ, Kim JW, Yim SV et al (2001) Fluoxetine enhances cell proliferation and prevents apoptosis in dentate gyrus of maternally separated rats. Mol Psychiatry 6:610. 10.1038/SJ.MP.4000954 11673802 10.1038/sj.mp.4000954

[34] Weaver ICG, Grant RJ, Meaney MJ (2002) Maternal behavior regulates long-term hippocampal expression of BAX and apoptosis in the offspring. J Neurochem 82:998–1002. 10.1046/J.1471-4159.2002.01054.X 12358805 10.1046/j.1471-4159.2002.01054.x

[35] Colle R, Segawa T, Chupin M et al (2017) Early life adversity is associated with a smaller hippocampus in male but not female depressed in-patients: A case-control study. BMC Psychiatry 17:1–9. 10.1186/s12888-017-1233-2 28202012 10.1186/s12888-017-1233-2 PMC5312536

[36] Zhang Y, Wang S, Hei M (2024) Maternal separation as early-life stress: Mechanisms of neuropsychiatric disorders and inspiration for neonatal care. Brain Res Bull 217:111058. 10.1016/J.BRAINRESBULL.2024.111058 39197670 10.1016/j.brainresbull.2024.111058

[37] Catz SD, Johnson JL (2001) Transcriptional regulation of bcl-2 by nuclear factor kappa B and its significance in prostate cancer. Oncogene 20:7342–7351. 10.1038/SJ.ONC.1204926 11704864 10.1038/sj.onc.1204926

[38] Wang C-Y, Guttridge DC, Mayo MW, Albert S, Baldwin J (1999) NF-κB Induces Expression of the Bcl-2 Homologue A1/Bfl-1 To Preferentially Suppress Chemotherapy-Induced Apoptosis. Mol Cell Biol 19:5923. 10.1128/MCB.19.9.5923 10454539 10.1128/mcb.19.9.5923 PMC84448

[39] Blanquie O, Kilb W, Sinning A, Luhmann HJ (2017) Homeostatic interplay between electrical activity and neuronal apoptosis in the developing neocortex. Neuroscience 358:190–200. 10.1016/J.NEUROSCIENCE.2017.06.030 28663094 10.1016/j.neuroscience.2017.06.030

[40] Dutcher EG, Pama EAC, Lynall M-E et al (2020) Early-life stress and inflammation: A systematic review of a key experimental approach in rodents. Brain Neurosci Adv 4:239821282097804. 10.1177/2398212820978049 10.1177/2398212820978049 PMC7780197 33447663

[41] Coccurello R, Bielawski A, Zelek-Molik A et al (2014) Brief maternal separation affects brain α1-adrenoceptors and apoptotic signaling in adult mice. Prog Neuro-Psychopharmacology Biol Psychiatry 48:161–169. 10.1016/j.pnpbp.2013.10.004 10.1016/j.pnpbp.2013.10.004 24128685

