# Supplementary material for: Long-Term Impact of Early-Life Stress on Hippocampal Apoptotic Gene Expression in BALB/c Mice
Source: Neurochem Res. 2026 Jun 25;51(4):203. doi: 10.1007/s11064-026-04822-7 (PMC13303556; doi:10.1007/s11064-026-04822-7)
Supplement: Supplementary file 1 — Supplementary Material 1. [file 11064_2026_4822_MOESM1_ESM.docx]

**Title of Manuscript :** Long-term Impact of Early-Life Stress on Hippocampal Apoptotic Gene Expression in *BALB/c* Mice

**Running Title :** Maternal Separation Alters Apoptosis Genes

**Authors’ information :** Aida Nurul Barokah^1^, İhsan Kıvanç Gürsoy^2^, Merve Hilal Dönmez^1,3^, Juliette Fitremann^4^, Arslan Bayram^5^, Keziban Korkmaz Bayram*^2,6^

1. Ankara Yıldırım Beyazit University, Institute of Health Science, Department of Translational Medicine, Ankara Türkiye.
2. Ankara Yıldırım Beyazit University, Faculty of Medicine, Department of Medical Genetics, Ankara Türkiye.
3. Technical University of Berlin, Faculty of Process Sciences, Institute of Biotechnology, Berlin, Germany
4. SOFTMAT, Université de Toulouse, CNRS UMR 5623, France
5. GENTAN, Genetic Diseases Evaluation Centre, İzmir Türkiye.
6. Izmir Biomedicine and Genome Centre, Gene Targeting and Transgenic Models Platform, İzmir Türkiye

*Corresponding author: Keziban Korkmaz Bayram; e-mail 1: [keziban.korkmazbayram@ibg.edu.tr](mailto:keziban.korkmazbayram@ibg.edu.tr); e-mail 2: [k.korkmaz.bayram@aybu.edu.tr](mailto:k.korkmaz.bayram@aybu.edu.tr); ORCID: 0000-0002-1228-1298

Aida Nurul Barokah ORCID: 0000-0003-3102-7858; e-mail: [biologistaida@gmail.com](mailto:biologistaida@gmail.com)

İhsan Kıvanç Gürsoy ORCID: 0009-0006-5393-4132; e-mail: [kivanc_gursoy@hotmail.com](mailto:kivanc_gursoy@hotmail.com)

Merve Hilal Dönmez ORCID: 0000-0001-6286-7038; e-mail: merve.isikli@outlook.de

Arslan Bayram ORCID: 0000-0002-3682-2140; e-mail: [dr.arslan.b@gmail.com](mailto:dr.arslan.b@gmail.com)

Juliette Fitremann ORCID: 0000-0002-1080-3742; e-mail: [juliette.fitremann@cnrs.fr](mailto:juliette.fitremann@cnrs.fr)

**Supplementary Methods**

**Tissue Collection and Hippocampal Dissociation into Single Cells**

*Tissue Collection:*

1. On postnatal day 35 (PND35), mice were sacrificed by cervical dislocation.
2. Brains were quickly removed, and the hemispheres were gently lifted toward the forebrain to expose the hippocampi.
3. The right and left hippocampi were carefully dissected using fine forceps.
4. Surgical instruments were disinfected with 75% ethanol between each dissection to prevent cross-contamination.
5. For RNA isolation, hippocampal tissues were snap-frozen in cryotubes using liquid nitrogen and stored at –80 °C.
6. For in vitro culture, hippocampi from control male mice were transferred into 15 mL Falcon tubes containing 5 mL of dissection solution and kept on ice for transport to the cell culture laboratory.

*Preparation of Dissection Solution:*

1. The dissection solution was prepared fresh by mixing the following components in a 50 mL Falcon tube:

- 500 mL Hank’s Balanced Salt Solution (HBSS)
- 5 mL penicillin/streptomycin (Gibco, Life Technologies)
- 5 mL of 1 M MgCl₂
- 3.5 mL of 1 M HEPES (pH 7.3; Lonza, Switzerland)
- 5 mL of 200 mM L-glutamine (Gibco, Life Technologies)

*Enzymatic Dissociation:*

1. The hippocampal tissues in dissection solution were centrifuged at 80 × *g* for 5 minutes at room temperature.
2. The supernatant was carefully discarded.
3. 1.5 mL of trypsin solution (prewarmed to 37 °C) was added to the pellet.
4. Samples were incubated at 37 °C for 10–20 minutes to enzymatically digest the tissue.

*Washing and Mechanical Dissociation:*

1. Following trypsinization, samples were centrifuged at 80 × *g* for 5 minutes, and the supernatant was discarded.
2. The pellet was washed twice with 5 mL of HBSS.
3. After the second wash, 1.5 mL of prewarmed culture medium (37 °C) was added.
4. The tissue pieces were gently triturated 20–30 times using a fire-polished Pasteur pipette to achieve a homogeneous single-cell suspension.
5. Trituration was repeated for 1 minute using a fresh fire-polished Pasteur pipette to ensure complete dissociation.

**Preparation of the Hydrogel**

1. For hydrogel preparation, 9 mg of GalC7 powder was weighed for each sample and placed into a clean glass vial.
2. 2 mL of nuclease-free water was added to the vial.
3. The vial was loosely capped and placed in a programmable oven preheated to 115 °C until the GalC7 was fully dissolved.
4. Once the solution was clear and homogenous, the hot solution was immediately transferred into pre-designated culture plate wells.
5. The plate was closed to prevent evaporation, and the temperature was gradually decreased to room temperature to allow for gelation.
6. Gels prepared one day in advance were saturated with culture medium and incubated overnight at 37 °C in a humidified incubator with 5% CO₂ prior to cell seeding.
